# Illustrating and Enhancing the Biosynthesis of Astaxanthin and Docosahexaenoic Acid in *Aurantiochytrium* sp. SK4

**DOI:** 10.3390/md17010045

**Published:** 2019-01-10

**Authors:** Jingrun Ye, Mengmeng Liu, Mingxia He, Ying Ye, Junchao Huang

**Affiliations:** 1 Key Laboratory of Economic Plants and Biotechnology, Yunnan Key Laboratory for Wild Plant Resources, Kunming Institute of Botany, Chinese Academy of Sciences, Kunming 650201, China; yejingrun@mail.kib.ac.cn (J.Y.); liumengmeng@mail.kib.ac.cn (M.L.); hemingxia@mail.kib.ac.cn (M.H.); yeying@mail.kib.ac.cn (Y.Y.); 2 University of Chinese Academy of Sciences, Beijing 100049, China

**Keywords:** *Aurantiochytrium*, Astaxanthin, Docosahexaenoic acid, genetic transformation

## Abstract

The marine thraustochytrids are a promising source of docosahexaenoic acid (DHA) and the ketocarotenoid astaxanthin. In this study, the biosynthetic pathways of these two important metabolites in *Aurantiochytrium* sp. SK4 was illustrated by the analyses of the genome, transcriptome, key enzymes, and pathway products. Two sets of genes were involved in two pathways for the biosynthesis of fatty acids. The absence of *Δ-15 desaturase* genes and the presence of docosapentaenoic acid (DPA), up to 12% of total fatty acids suggest that *Aurantiochytrium* sp. SK4 may synthesize DHA mainly via a polyketide synthase (PKS) pathway. Three enzymes, namely geranyl diphosphate synthase (GPPS), farnysyl diphosphate synthase (FPPS), and geranylgeranyle diphosphate synthase (GGPPS) were found to be involved in the formation of GGPP that was subsequently catalyzed to β-carotene by a trifunctional CrtIBY enzyme. β-Carotene might be ketolated and then hydroxylated into astaxanthin based on the carotenoid profiles. The formation of GGPP was proposed to be the limiting steps for carotenoid production. Overexpression of the *Archaeoglobus* GPS together with the *Escherichia coli* isopentenyl pyrophosphate isomerase, and *Vitreoscilla* hemoglobin resulted in not only 1.85- and 5.02-fold increases of total carotenoids and astaxanthin, but also 2.40- and 2.74-fold increases of total fatty acids and DHA. This study provides insights into the biosynthesis of carotenoids and fatty acids in *Aurantiochytrium*.

## 1. Introduction

Thraustochytrids are heterotrophic marine microbes with the ability to accumulate high levels of docosahexaenoic acid (DHA, 22:6 n-3) [1,2]. DHA is a key omega-3 polyunsaturated fatty acids (PUFAs), which has received worldwide attention due to its beneficial effects for humans [3,4,5]. The biosynthesis of DHA was proposed to be mainly mediated via the polyketide synthase (PKS) pathway [6,7,8]. Currently, the primary commercial source of DHA is marine fish oils with the disadvantages of low DHA levels, unstable quality, high processing costs, undesirable smell, unsustainability, and environmental contaminants [9,10,11,12]. Some *Aurantiochytrium* strains have been recognized and established as an alternative source for commercial DHA production because of their high growth rates and ability to accumulate high levels of DHA [13,14,15].

Certain strains of *Aurantiochytrium* also produce such carotenoids as astaxanthin and cantaxanthin [16,17,18]. Astaxanthin (3,3′-dihydroxy-β-carotene-4,4′-dione) is only biosynthesized by a limited number of organisms via the ketolation and hydroxylation of β-carotene. This red ketocarotenoid has strong antioxidant activity and therefore has been widely applied in aquaculture, nutraceutical, pharmaceutical, and cosmetic industries [19]. Up to date, astaxanthin is mainly obtained by chemical synthesis and extraction from *Haematococcus pluvialis* [20,21]. The astaxanthin produced by chemical synthesis contains four different stereoisomer configurations: (3S, 3′S), (3S, 3′R), (3R, 3′S) and (3R, 3′R). The proportion of functional (3S, 3′S) isomer were only 25% [22]. In consideration of the advantage of astaxanthin and pathogenicity of synthetic products [23] such as synthetic β-carotene [24], increasing attention focused on natural astaxanthin. The strict growth conditions and quite low yields of *H. pluvialis* have limited the application of natural astaxanthin.

*Aurantiochytrium* sp. SK4 belongs to Thraustochytrids that has the ability to accumulate high amounts of DHA as well as significant contents of carotenoids including astaxanthin [17]. Furthermore, by overexpressing an oxygen-involved protein, this microbe produced much higher amounts of fatty acids and astaxanthin [17], suggesting the potential of the organism to serve as a cell factory for producing such important metabolites as astaxanthin and DHA by overexpressing rate-limiting enzymes. However, the influence on the biosynthesis of DHA and astaxanthin remains largely unknown, which limits the metabolic engineering of the microbe for high-yield production of the metabolites.

In this study, the biosynthetic pathways of astaxanthin and DHA in *Aurantiochytrium* sp. SK4 was illustrated by characterizing the genes involved in the pathways through the analyses of the genome, transcriptome, gene functions, and metabolites. Moreover, the biosynthesis of carotenoids and fatty acids in *Aurantiochytrium* was greatly enhanced by directing farnesyl pyrophosphate (FPP) to geranylgeranyl pyrophosphate (GGPP), the key limiting step to carotenoid production in the organism, via the expression of the *Archaeoglobus GPS* with the ability to convert isopentenyl pyrophosphate (IPP) and dimethylallyl diphosphate (DMAPP) into GGPP.

## 2. Result and Discussion

### 2.1. Analysis of Aurantiochytrium sp. SK4 Genome and Transcriptome

To provide a genomic architecture of *Aurantiochytrium* sp. SK4, two DNA libraries were constructed and sequenced using the Illumina HiSeq2000 platform (San Diego, USA). The assembled genome size of SK4 was 49.62 Mb by k-mer-based estimation. There were 20,098 coding genes mapped on the genome.

The genome features of *Aurantiochytrium* sp. SK4 were compared with five green algae (*Chlamydomonas reinhardtii* [25], *Chlorella* sp. NC64A [26], *Chromochloris zofingiensis* [27], *Coccomyxa subellipsoidea* C-169 [28], *Monoraphidium neglectum* [29]) and the model plant *Arabidopsis thaliana* [30] (Table S1). The genome of *Aurantiochytrium* sp. SK4 consists of the lowest average intron numbers (1.4 introns per transcript) and 45.5% of its coding genes have no introns. By contrast, more than 80% of the genes from the five algal genomes and 76% from Arabidopsis thaliana contain at least one intron. In addition, *Aurantiochytrium* sp. Sk4 genome demonstrated a modest GC content (56.7%) and low repeat sequences (Table S1).

To reinforce the annotation of the genomic data, two cDNA libraries of *Aurantiochytrium* sp. SK4 cells cultivated for 24 hours and 96 hours were constructed, sequenced, and analyzed. Overall, a total of 27,251 uni-genes were identified, which ranged from 201 bp to 29,208 bp, with an average length of 758 bp. The length distributions of the assembled uni-genes are shown in Figure S1. There were 9829 uni-genes those were longer than 1000 bp. The uni-genes were aligned to five different databases including NR, Swiss-Port, GO, COG, and KEGG using BLAST (https://blast.ncbi.nlm.nih.gov/Blast.cgi) with an *E*-value threshold of 1e-05. Of the uni-genes, 15,812 (58.0%) showed significant matches with genes from at least one database (Figure S2).

Differentially expressed genes (DEGs) were identified. A total of 2069 DEGs (1319 up-regulated and 750 down-regulated) were found between the cells cultivated for 24 hours and 96 hours by KEGG (https://www.kegg.jp/kegg/pathway.html) pathway enrichment (*p* value <0.01), these DEGs were shown to be focused on "Terpenoid backbone biosynthesis", "Ubiquinone, and other terpenoid-quinone biosynthesis", "Steroid biosynthesis", "Valine, leucine and isoleucine degradation", "Pyruvate metabolism", and some more functions (Figure S3). Most of these pathways were related to carotenoid and fatty acid biosynthesis. Thus, *Aurantiochytrium* sp. Sk4 cells underwent great changes in gene expression during the biosynthesis of carotenoids and fatty acids.

### 2.2. Illustrating the Biosynthetic Pathway of DHA

**Figure 1:**
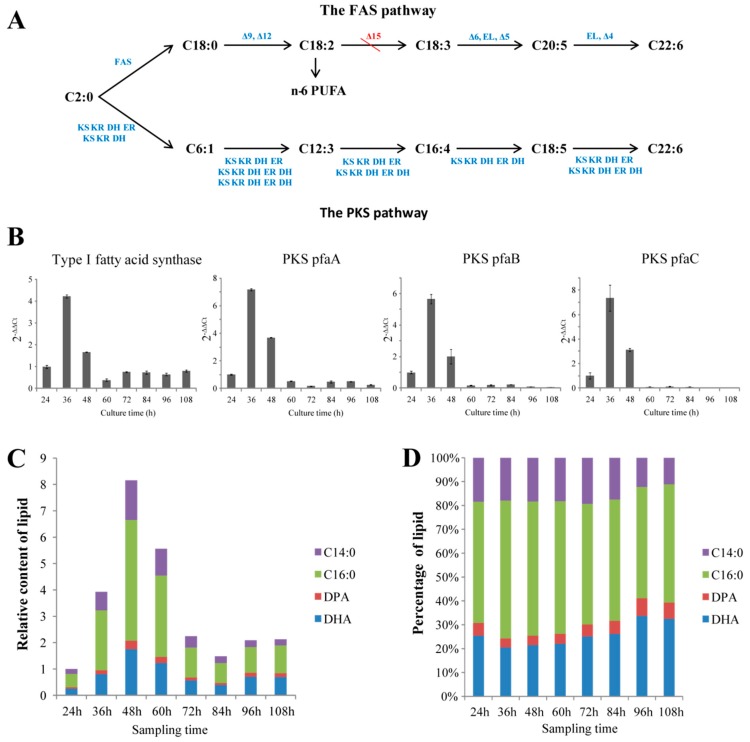
Overview of the fatty acid information in SK4. Fatty acid biosynthesis pathways (**A**), the expression trends of Type I fatty acid synthase and three subunits in the polyketide synthase (PKS) pathway (**B**), relative fatty acid content (**C**) and percentage (**D**) of C14:0, C16:0, docosapentaenoic acid (DPA) and docosahexaenoic acid (DHA). *ω*-*3*: *ω*-3 desaturase; EL: elongase; KS: 3-ketoacyl synthase; KR: 3-ketoacyl-ACP reductase; ER: enoyl reducatase; DH: dehydrase/isomerase. Data are shown as mean ± SD, n = 3.

**Figure 2:**
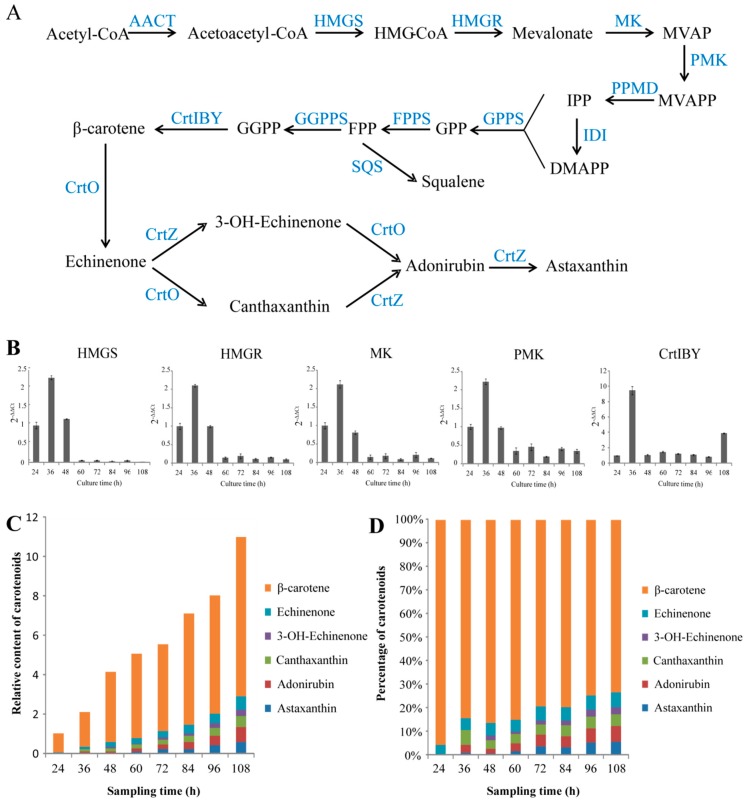
The information on carotenoid biosynthesis in Aurantiochytrium sp. SK4. Putative biosynthetic pathways of carotenoids (**A**), the expression trends of 3-hydroxy-3-methylglutaryl-CoA synthase (HMGS), 3-hydroxy-3-methylglutaryl-CoA reductase (HMGR), mevalonate kinase (MK), phosphomevalonate kinase (PMK), and CrtIBY (**B**), relative carotenoid content (**C**) and percentage (**D**). SQS, squalene synthesis. Data are shown as mean ± SD, n = 3.

Thraustochytrids were proposed to use two pathways fatty acid synthase (FAS) and PKS pathways) to synthesize fatty acids [8,31,32]. Genomic and transcriptomic mining revealed that *Aurantiochytrium* sp. SK4 consisted of almost all the genes for the two pathways. The candidate genes encoding for the enzymes involved in *FAS* pathway included a type I fatty acid synthase (*FAS*), 6 fatty acid desaturases (*FAD*: *Δ-9, Δ-12*, *Δ-6*, *Δ-5*, *ω-3*, and *Δ-4 desaturases*), and 4 fatty acid elongases (Figure 1A). Transcriptomic data showed that these genes remarkably expressed except for the *Δ-9*, *Δ-6 and* ω-*3 desaturases* (Table S2). Interestingly, no *Δ-15 desaturase* genes were found in both the genome and transcriptome sequences. The genes (*pfaA*, *pfaB* and *pfaC*) encoding for the subunits of the polyketide synthase (PKS) were identified, which expressed at moderate levels according to the transcriptomic data (Figure 1A, Figure S4). To further correlate the accumulation of fatty acids with the expression of the key genes involved in the FAS and PKS pathways, the transcription of one FAS and three PKS genes were detected at eight different culturing times by quantitative real-time PCR (qRT-PCR). The transcription trends of the four genes increased rapidly at the logarithmic phase but decreased at stationary phase and decline phase (Figure 1B). Gas chromatography-mass spectrometry (GC-MS) detection revealed four main fatty acids, i.e. myristic acid (C14:0), palmitic acid (C16:0), docosapentaenoic acid (DPA) and DHA. DHA and palmitic acid were the predominant fatty acids (Figure 1C,D). The contents of total lipids and DHA increased and reached a maximum at 48 hours and thereafter decreased during the decline phase (Figure 1C,D). Thus, the expression of the genes correlated well with the accumulation of fatty acids. The presence of DPA and the absence of *Δ-15 desaturase* genes suggest *Aurantiochytrium* sp. SK4 might synthesize DHA via the PKS pathway rather than the FAS pathway. The growth curve of *Aurantiochytrium* sp. SK4 of Figure 1 and Figure 2 were shown in Figure S5.

### 2.3. Illustrating the Biosynthetic Pathway of Astaxanthin

The candidate genes encoding the enzymes for the biosynthesis of astaxanthin were mined from the genome and transcriptome data. The mevalonate (MVA) pathway was found to be involved in the formation of isopentenyl pyrophosphate (IPP) (Figure 2A). The most genes involved in the MEP pathway were not found in transcriptomic data. Three enzymes were predicted to catalyze the formation of GGPP from IPP and DMAPP: Geranyl diphosphate synthase (GPPS), farnesyl diphosphate synthase (FPPS), and geranylgeranyl diphosphate synthase (GGPPS). The genes encoding for the enzymes of phytoene synthase (CrtB), phytoene desaturase (CrtI), and lycopene cyclase (CrtY), which are involved in the biosynthesis of β-carotene from GGPP, were found to be consecutively fused into an open reading frame without stop codons (*CrtIBY*). Similar *CrtIBY* genes were also found in five other *Aurantiochytrium* species, one of which was confirmed to catalyze β-carotene formation from GGPP [33]. The functional domains of CrtIBY of *Aurantiochytrium* sp. SK4 and the alignment of different CrtIBY protein sequences were showed in Figure S6. A typical β-carotene hydroxylase (CrtZ) gene and three β-carotene ketolase (CrtO) genes were identified, which might be involved in converting β-carotene into astaxanthin. To reveal the regulation of astaxanthin biosynthesis at gene expression trends, the transcriptional patterns of five genes involved in the MVA pathway including 3-hydroxy-3-methylglutaryl coenzyme A synthase (*HMG-CoA synthase*), 3-hydroxy-3-methylglutaryl-coenzyme A reductase (*HMG-CoA reductase*), mevalonate kinase (*MVA kinase*), phosphomevalonate kinase (*PMVA kinase*) and the *CrtIBY* involved in the conversion of GGPP to β-carotene were detected at eight different time points by qRT-PCR (Figure 2B). All of the five genes reached expression peak at 36 h. The MVA pathway genes were upregulated at the early stages of cell development, but down-regulated when the cells were in the stationary phase. In contrast, *CrtIBY* kept a relatively stable expression trend at stationary and decline phases. To further correlate the expression of the genes with the carotenoid composition, the dynamic evolution of carotenoids were analyzed. *Aurantiochytrium* sp. SK4 was found to consist of five main carotenoids: β-carotene, echinenone, 3-OH echinenone, canthaxanthin, adonirubin (phenicoxanthin), and astaxanthin (Figure 2C,D). β-Carotene and canthaxanthin were the major carotenoids, with the other three as minor ones. Since β-carotene was the predominant carotenoid (>60% of total carotenoids), the ketolation and hydroxylation of it should be the limiting steps for astaxanthin formation. The biosynthesis of astaxanthin in *Aurantiochytrium* sp. SK4 was proposed as shown in Figure 2A. Previously, overexpression of heterologous hemoglobin in *Aurantiochytrium* was found to enhance astaxanthin production [17], indicating that this microorganism could be manipulated to further enhance the production of astaxanthin and possibly other metabolites, such as DHA and carotenoids by the expression of some rate-limiting enzymes.

### 2.4. Metabolic Engineering of Aurantiochytrium sp. SK4 for Carotenoid Production

**Figure 3:**
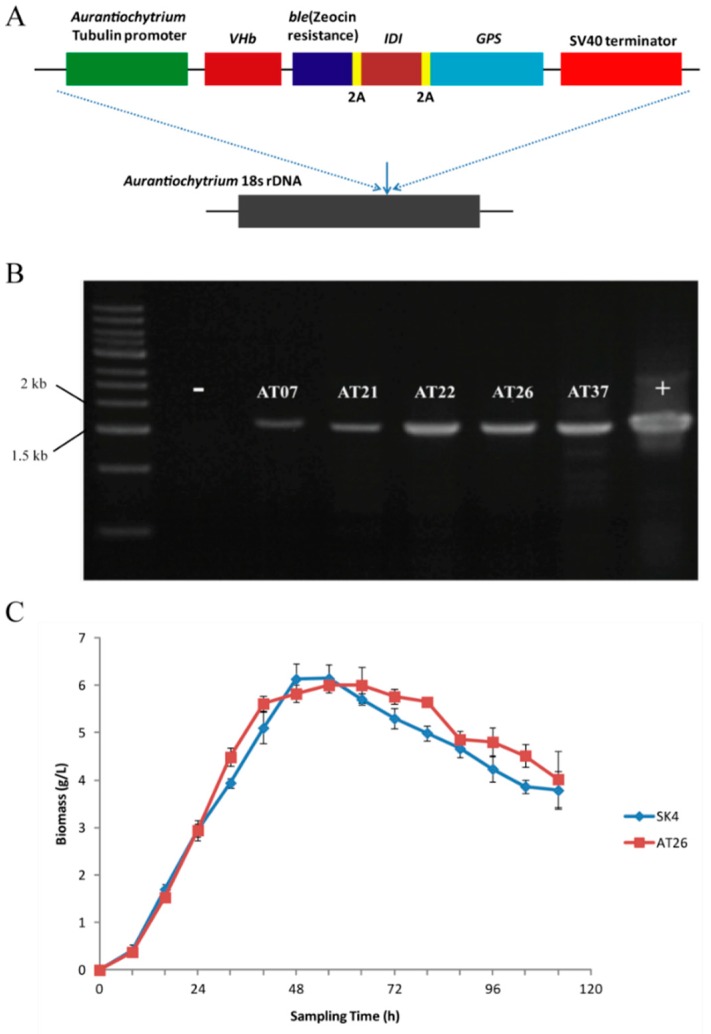
The information of transformant AT26. (**A**) Schematic diagram of the p-VBIG construct containing the endogenous tubulin promotor, −*VHb*−*ble*-2A-*IDI*-2A-*GPS* (VBIG) genes, and the SV40 terminator. (**B**) Genomic PCR detection of VBIG in the transformants resistance to zeocin. (**C**) Growth curves of *Aurantiochytrium* sp. SK4 and its transformant AT26. Data are shown as mean ± SD, n = 3.

The low carotenoid contents of *Aurantiochytrium* sp. SK4 could result from insufficient GGPP precursor for β-carotene formation due to the competition of FPP for squalene formation (Figure 2A). There is substantial squalene (>10 mg × g^−1^) which consumes much FPP (Table S3) that as the intermediates of IPP to GGPP was the precursor of carotenoids. The *Archaeoglobus* homologous enzyme (GPS) was demonstrated to effectively catalyze GGPP synthesis from IPP and GGPP to produce high amounts of β-carotene. [34,35]. Thus we intended to express the *Archaeoglobus GPS* in *Aurantiochytrium* sp. SK4 thus as to further investigate the biosynthesis of carotenoids. The *GPS* was linked downstream to an *E. coli IDI* gene via a 2A sequence [36], which was further inserted into the *Aurantiochytrium* expression construct [17]. The resulting vector was named as p-VBIG (Figure 3A). The linear DNA fragments of p-VBIG were introduced into *Aurantiochytrium* sp. SK4 by an electroporation approach. The *ble* gene conferring zeocin resistance was used as a selectable marker to screen putative transformants. Zeocin-resistant transformants were further detected by PCR to confirm the presence of the transgenes (Figure 3B). One transformant (AT26) was selected for further investigation. The transgenes seemed not to disturb the growth of AT26 as demonstrated by the similar growth curves of wild type (WT) and the transformant (Figure 3C). 

**Figure 4:**
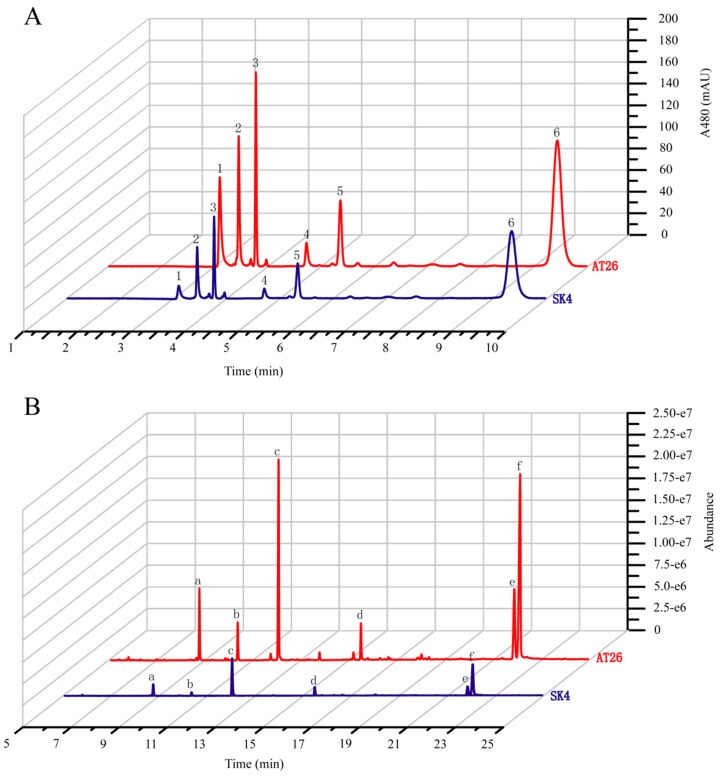
Analysis of carotenoids and fatty acids from the wild type (SK4) and VBIG-expressing *Aurantiochytrium* sp. SK4 (AT26). (**A**) Ultra-Performance liquid chromatography (UPLC) chromatograms of WT and AT26 sampling at 120 hours. 1. Astaxanthin, 2. Adonirubin, 3. Canthaxanthin, 4. 3-OH-Echinenone, 5. Echinenone, 6. β-carotene. (**B**) Gas chromatography-mass spectrometry (GC-MS) chromatograms of wild type (WT) and AT26 sampling at 72 hours. a. C14:0, b. C15:0; c. C16:0, d.C18:0, e. DPA, f. DHA. The blue line represents SK4 and the red line represent AT26.

However, the transgenes greatly enhanced the biosynthesis of both carotenoids and fatty acids (Figure 4, Table 1). AT26 produced 1.78-fold total carotenoids of WT, with β-carotene the predominant carotenoid (66.9% of total carotenoids). In addition, AT26 accumulated 5.02-fold astaxanthin of WT, possibly resulting from the expression of *VHb* as shown by Suen et al. [17]. The transformant AT26 produced 141.1 ± 6.5 µg × g^−1^ astaxanthin which was much higher than *Schizochytrium* sp. SH104 and its mutant SHG104 [37]. However, astaxanthin only accounted for 7.87% of total carotenoids (Table 1), suggesting that the ketolation and hydroxylation of β-carotene are the limiting steps for astaxanthin formation. If this bottleneck can be removed, the astaxanthin production might be increased to a level that has the potential to meet the market demand. The increased production of carotenoids might result from the cooperation of the *VHb*, *IDI*, and *GPS*, which could decrease the FPP levels and hence decrease the production of squalene. We detected the contents of squalene in WT and AT26 and found that the transformant produced squalene at the amount of less than 1/20 of WT (Table S3). To investigate if the biosynthesis of fatty acids is coupled with carotenoids, we analyzed the fatty acid compositions and contents in AT26. Interestingly, the expression of VBIG triggered great increases of total fatty acids and PUFA, especially DHA both in contents and proportions (Figure 4B and Table 1). The contents of total fatty acids and DHA in AT26 were 2.40-, and 2.74-fold of WT. These results supported that the biosynthesis of carotenoids and fatty acids might be coordinated as reported previously [38,39]. The overexpression of *VHb* was previously found to enhance the production of astaxanthin at the expense of β-carotene, echinenone, and canthaxanthin in *Aurantiochytrium* sp. SK4 [17]. In contrast, the co-expression of the *GPS*, *IDI* and *VHb* greatly enhanced the contents of both total carotenoids and fatty acids.

## 3. Material and Methods

### 3.1. Strain and Culture Conditions

*Aurantiochytrium* sp. SK4 was isolated from Hong Kong [17] and was maintained on glucose-yeast extract agar plates (5 g/L glucose, 1 g/L yeast extract, 50% artificial seawater (*v*/*v*), 1% agarose, pH 6). The seed culture medium contained 5 g/L glucose, 2 g/L yeast extract and 50% artificial seawater. For the quantitative analyses of carotenoids and fatty acids, SK4 cells were cultured in 50 mL glucose-yeast extract medium in 250 mL Erlenmeyer flasks, which contained 50% artificial seawater with 20 g/L glucose and 4 g/L yeast extract, pH 6. The flasks were incubated at 150 rpm, 25 °C in dark conditions. The artificial seawater consists of (per liter) 30 g NaCl, 2.638 g MgSO_4_, 0.7 g KCl, 0.756 g CaCl_2_ and 10.8 g MgCl_2_·6H_2_O.

### 3.2. DNA Amplification, Cloning, and Sequencing

Genomic DNA was isolated by a standard cetyltrimethylammonium bromide (CTAB) protocol. The genome of SK4 was sequenced using the Illumina HiSeq platform (Illumina, San Diego, CA, USA). Two paired-end DNA libraries of Illumina HiSeq were prepared (250bp and 500bp). Following quality clipping and adapter trimming, genome sequence assembly was achieved by the combination of SOAPdenovo v2.04 and GapCloser v1.12 [40]. RepeatMasker version 4.0.3 [41] was used to identify repeat sequences. Gene prediction was carried out by Maker2 software [42] and the gene sequences were queried against the National Center for Biotechnology Information (NCBI) non-redundant protein sequences (Nr), GENES, STRING and GO using blastp software (v2.2.28+, https://blast.ncbi.nlm.nih.gov/Blast.cgi?CMD=Web&PAGE_TYPE=BlastDocs&DOC_TYPE=Download).

### 3.3. RNA Extraction and RNA-Seq

Total RNA of *Aurantiochytrium* sp. SK4 was isolated using TRIzol reagent following the manufacturer’s instructions. mRNA was extracted from the total RNA using NEBNext Poly(A) mRNA Magnetic Isolation Module (NEB, Ipswich, MA, USA). cDNA synthesis was performed by NEBNext mRNA Library Prep Master Mix Set for Illumina (NEB, Ipswich, MA, USA) and NEBNext Multiplex Oligos for Illumina (NEB, Ipswich, MA, USA) according to the standard protocol of Illumina sample preparation method.

Paired-end sequencing of cDNA was carried out with the Illumina Hiseq2500 platform (Illumina, San Diego, CA, USA). The reads were assembled into contiguous sequences (contigs) by Trinity software [43]. All the non-redundant contigs were used for gene prediction by GetORF software (http://genome.csdb.cn/cgi-bin/emboss/help/getorf). For transcriptome annotation, the final sequences were queried against the NCBI non-redundant protein sequences (Nr), SwissProt, GO, COG, and KEGG using NCBI blast software with a cut off E-value ≤ 10−5. Raw sequence reads of genome and transcriptome have been deposited into the NCBI Sequence Read Archive (https://submit.ncbi.nlm.nih.gov/subs/sra/) under the accession number SRR8029982 and SRR8029983.

### 3.4. Differential Expression Analysis

Expression levels of uni-genes were calculated based on RPKM (reads per kilobase transcriptome per million mapped reads) and separate sequence read datasets were used as inputs into the DESeq R package (http://bioconductor.org/packages/release/bioc/html/DESeq.html)) to analyze the uni-genes expression. Differential expression analysis (fold changes) and related statistical computations of the two conditions were conducted using the EBSeq R package (https://bioconductor.org/packages/release/bioc/html/EBSeq.html)). The resulting *p*-values were adjusted using Benjamini-Hochberg approach for controlling the false discovery rate (FDR). Genes with an adjusted FDR < 0.05 and | log2 (RPKM_Sample_/PRKM_Control_) | > 1 were classified as differentially expressed. Gene set enrichment analysis was carried out using DEGs by fisher’s exact text and adjusted using Benjamini approach to identify functional GO terms and KEGG pathways.

### 3.5. Quantitative Real-Time PCR

Quantitative real-time PCR (qRT-PCR) was performed to explore the transcription of genes involved in astaxanthin and DHA biosynthesis and also to validate the RNA-Seq results. Total RNA was extracted from eight different time points of cell cultivation (24, 36, 48, 60, 72, 84, 96 and 108 hours). RNA samples were treated with RNase-free DNase I (TaKaRa Biotech Co., Ltd., Dalian, China) to remove any contaminating DNA. Agarose gel electrophoresis (1.5%), Nanodrop 2000 (Thermo Scientific, Wilmington, USA) and Agilent 2100 Bioanalyzer (Agilent Technologies, Santa Clara, USA) were used to check RNA integrity, concentration, and quality. A total of 2.5 µg RNA was used for the first-strand cDNA synthesis in a volume of 20 µL reaction, using Prime Script II 1st Strand cDNA Synthesis kit (TaKaRa Biotech Co., Ltd., Dalian, China). The cDNA synthesized was diluted 15-folds before being used. qRT-PCRs were performed on a CFX Connect Real-Time System (Bio-Rad, Hercules, CA, USA) with a 20 µL reaction volume consisting of 0.25 mM of each primer (Table S4), 10 µL of iTaq SYBR Green Supermix (Bio-Rad) and 2 µL of template cDNA. The PCR profile was: 30 seconds at 95 °C followed by 40 cycles of 5 seconds at 95 °C, 15 seconds at 60 °C and followed by 0.5 °C increment at 5 seconds/step from 65 to 95 °C for the Melt Curve analysis. Actin gene was set as reference gene and the 2^−ΔΔCT^ method was used to calculate the relative changes in gene expression. The data obtained was analyzed using CFX Manager™ Software v3.1 (Bio-Rad).

### 3.6. Carotenoid Analysis

Carotenoid analysis was performed according to that described by Suen et al. [17]. Carotenoid extracts were analyzed with an Aglient Ultra-Performance liquid chromatography (UPLC) 1290 Infinity, which was equipped with an Aglient Eclipse plus C18 RRHD 1.8 µm column (2.1 mm × 50 mm) (Agilent, Santa Clara, CA, USA). The mobile phase consisted of solvent A (20% water, 60% acetonitrile, 5% isopropanol and 15% methanol) and solvent B (80% acetonitrile, 5% isopropanol and 15% methanol). The extracted pigments were eluted at a flow rate of 0.5 mL/min with the following process: 100% A for 1 minute; a liner gradient from 0 to 100% B within 1 minute; 100% B for 4 minutes. The carotenoid contents were detected at 480 nm wavelength. The carotenoids were qualified and quantified by comparing the retention times and peak areas of the individual standard. The carotenoids standard curves were made for determining the amounts of carotenoids. The analyses were carried out in duplicates for each sample.

### 3.7. Lipid Extraction and Fatty Acid Analysis

Lipid extraction was carried out according to the Folch method [44]. Direct trans-esterification of dried samples was performed for determining the content of fatty acids. The fatty acid methyl esters (FAME) were analyzed using a gas chromatography-mass spectrometry (GC-MS) machine (Agilent Technologies 7890/5975) equipped with a DB-5 (30 m × 250 μm × 0.25 μm, Agilent) column (Agilent, Santa Clara, CA, USA). Helium was used as carrier gas (constant flow: 1.2 mL/min), the injector was kept at 250 °C with an injection volume of 1 μL under a split ratio of 20:1, and the column temperature was first set at 80 °C for one minute and then increased at a rate of 10 °C per minute to 250 °C, the rate was then changed to 8 °C per minute until 280 °C was reached and maintained for 5 minutes. Identification of individual FAME was based upon comparing the mass to charge ratio with that of standard FAME mixtures and NIST database.

### 3.8. Construction of Plasmid p-VBIG and Transformation

The plasmid p-VBIG was constructed by inserting a 2A-*IDI*-2A-*GPS* DNA fragment into the pUC18-VHb-ble vector [17] by Gibson Assembly Master Mix (NEB, Ipswich, MA, USA). The schematic diagram of p-VBIG is shown in Figure 3A. *Aurantiochytrium* sp. SK4 was transformed with p-VBIG by electroporation using a GenePulser Xcell™ (Bio-Rad, Hercules, CA, USA). The parameters of electroporation were set as 2 kV, 50 μF, and 300 Ω. Electroporated cells were resuspended in 50 mL of culture medium and cultivated for 16 hours in an orbital shaker at 150 rpm and 25 °C in the dark. The cells were then spread on agar plates containing 50 μg/mL zeocin as a selective agent. The colonies resistant to zeocin were isolated and cultured for subsequent analysis. The integration of transgenes was verified by PCR using primers VBIGF and VBIGR (Table S4).

### 3.9. Statistical Analysis

All experiments had three biological replications and at least two measurement replicates. Data were expressed as means ± SD (Standard deviation) and analyzed using IBM SPSS Statistics 25 (IBM, Armonk, NY, USA).

## 4. Conclusions

The draft genome and transcriptome of *Aurantiochytrium* sp. SK4 were analyzed to illustrate the biosynthesis of astaxanthin and DHA in the biotechnologically important microorganism. The formation of GGPP from IPP and astaxanthin from β-Carotene might be the limiting steps for carotenoid accumulation in *Aurantiochytrium* sp. SK4. The organism was genetically manipulated to enhance the production of astaxanthin and DHA by the expression of an efficient *GPS* together with an *IDI* and *VHb*. This study opens the door for *Aurantiochytrium* strains to more efficiently produce important metabolites by metabolic engineering of the pathways.

## Figures and Tables

**Table 1:** Contents and compositions of carotenoids and fatty acid in wild type SK4 and its transformant AT26.

		Sk4	AT26
**Carotenoid content μg × g^−1^ DCW (% composition) ^A^**	Astaxanthin ^1^	28.2 ± 2.9 (2.9%)	141.1 ± 6.5 (7.9%)
	Adonirubin ^2^	65.3 ± 4.1 (6.7%)	133.5 ± 0.1 (7.4%)
	Canthaxanthin ^3^	72.6 ± 5.5 (7.5%)	156.1 ± 12.1 (8.7%)
	3-OH-Echinenone ^4^	20.0 ± 0.6 (2.1%)	39.9 ± 1.1 (2.2%)
	Echinenone ^5^	70.4 ± 5.0 (7.3%)	122.4 ± 7.0 (6.8%)
	β-carotene ^6^	712.7 ± 51.0 (73.5%)	1200.5 ± 73.1 (66.9%)
	Total	969.2 ± 67.5	1793.5 ± 86.8
**Fatty acid content mg × g^−1^ DCW (% composition) ^B^**	C14:0 ^a^	16.4 ± 1.2 (9.1%)	25.1 ± 3.6 (5.8%)
	C15:0 ^b^	8.4 ± 0.3 (4.6%)	16.0 ± 1.8 (3.7%)
	C16:0 ^c^	47.4 ± 2.8 (26.2%)	91.4 ± 8.2 (21.0%)
	C16:1	0.0 ± 0.0 (0.0%)	6.7 ± 0.5(1.5%)
	C17:0	0.0 ± 0.0 (0.0%)	6.8 ± 0.3 (1.6%)
	C18:0 ^d^	14.3 ± 0.7 (7.9%)	20.4 ± 0.3 (4.7%)
	C18:1	0.0 ± 0.0 (0.0%)	6.9 ± 0.1 (1.6%)
	C20:4 (AA)	0.0 ± 0.0 (0.0%)	5.2 ± 0.1 (1.2%)
	C20:5 (EPA)	5.9 ± 0.1 (3.3%)	6.4 ± 0.1 (1.5%)
	C22:5 (DPA) ^e^	21.8 ± 1.1 (12.0%)	69.2 ± 3.1 (15.9%)
	C22:6 (DHA) ^f^	64.2 ± 3.5 (35.4%)	175.8 ± 7.2 (40.5%)
	others	2.7 ± 3.8 (1.5%)	5.0 ± 0.1 (1.2%)
	SFA	86.6 ± 5.0 (47.8%)	159.8 ± 14.2 (36.8%)
	MUFA	0.0 ± 0.0 (0.0%)	13.1 ± 0.5 (3.0%)
	PUFA	91.8 ± 4.6 (50.7%)	256.5 ± 10.5 (59.0%)
	TFA	181.1 ± 4.1	434.4 ± 4.1

DCW, dry cell weight; AA, arachidonic acid; EPA, eicosapentaenoic acid; DPA, docosapentaenoic acid; DHA, docosahexaenoic acid. SFA, saturated fatty acids; MUFA, monounsaturated fatty acids; PUFA, polyunsaturated fatty acid; TFA, total fatty acids. ^A^ Carotenoid content after 120 hours of shaker culture. ^B^ Fatty acid content after 72 hours of shaker culture. ^1–6^ and ^a–f^ were the peak assigned in Figure 4. Data are shown as mean ± SD, n = 3.

## Supplementary Materials

Supplementary materials associated with this article can be found in the online version, http://www.mdpi.com/1660-3397/17/1/45/s1. Figure S1: Unigene length distribution. Figure S2: Functional annotation of assembled unigenes in *Aurantiochytrium* sp. SK4. Figure S3: KEGG pathway enrichment analysis of DEGs. Figure S4: Genes encoding enzymes of PKS pathway in *Aurantiochytrium* sp. SK4. Figure S5: The growth curve of *Aurantiochytrium* sp. SK4 of Figure 1 and Figure 2. Figure S6: The conseved domains of CrtIBY and alignment of amino acid sequences of different CrtIBY. Table S1: Comparison of *Aurantiochytrium* sp. SK4 Genome Statistics to other five algae and Arabidopsis thaliana genome. Table S2: The expression of genes about carotenoids and fatty acid biosynthesis in transcriptome. Table S3: Contents of squalene in wild-type SK4 and the transformant AT26. Table S4: Primers Used for qRT-PCR and the detection of the p−VBIG.
