# Supplementary material for: Illustrating and Enhancing the Biosynthesis of Astaxanthin and Docosahexaenoic Acid in Aurantiochytrium sp. SK4
Source: Mar Drugs. 2019 Jan 10;17(1):45. doi: 10.3390/md17010045 (PMC6357005; doi:10.3390/md17010045)
Supplement: Supplementary file 1 [file marinedrugs-17-00045-s001.pdf]

Supplementary Material

Defining and enhancing the biosynthesis of astaxanthin and Docosahexaenoic acid in *Aurantiochytrium* sp. SK4

Jingrun Ye[a, b], Mengmeng Liu[a, b], Mingxia He[a], Ying Ye[a,b], Junchao Huang[a*]

[a]Key Laboratory of Economic Plants and Biotechnology, Yunnan Key Laboratory for Wild Plant Resources, Kunming Institute of Botany, Chinese Academy of Sciences, Kunming 650201

[b]University of Chinese Academy of Sciences, Beijing 100049, People's Republic of China

***Corresponding author**

Email: huangjc@mail.kib.ac.cn.

Phone: +86-875-65228058

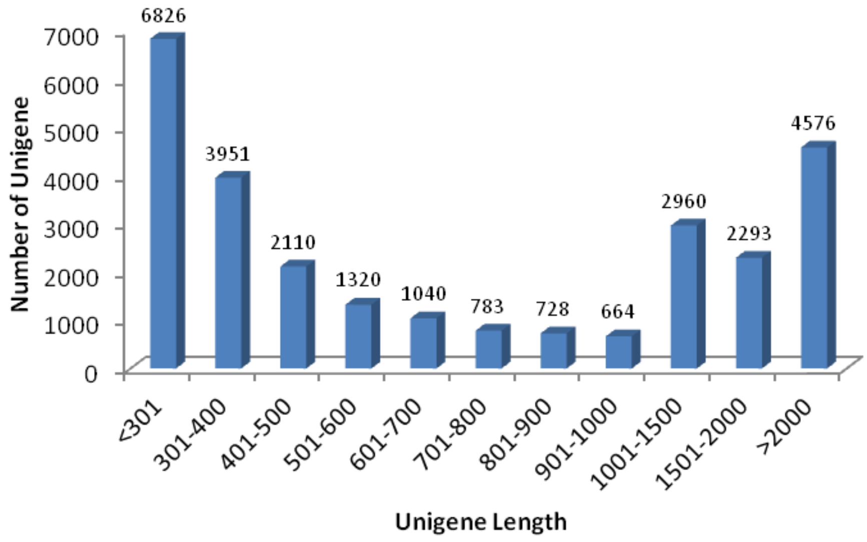

Figure S1. Uni-gene length distribution.

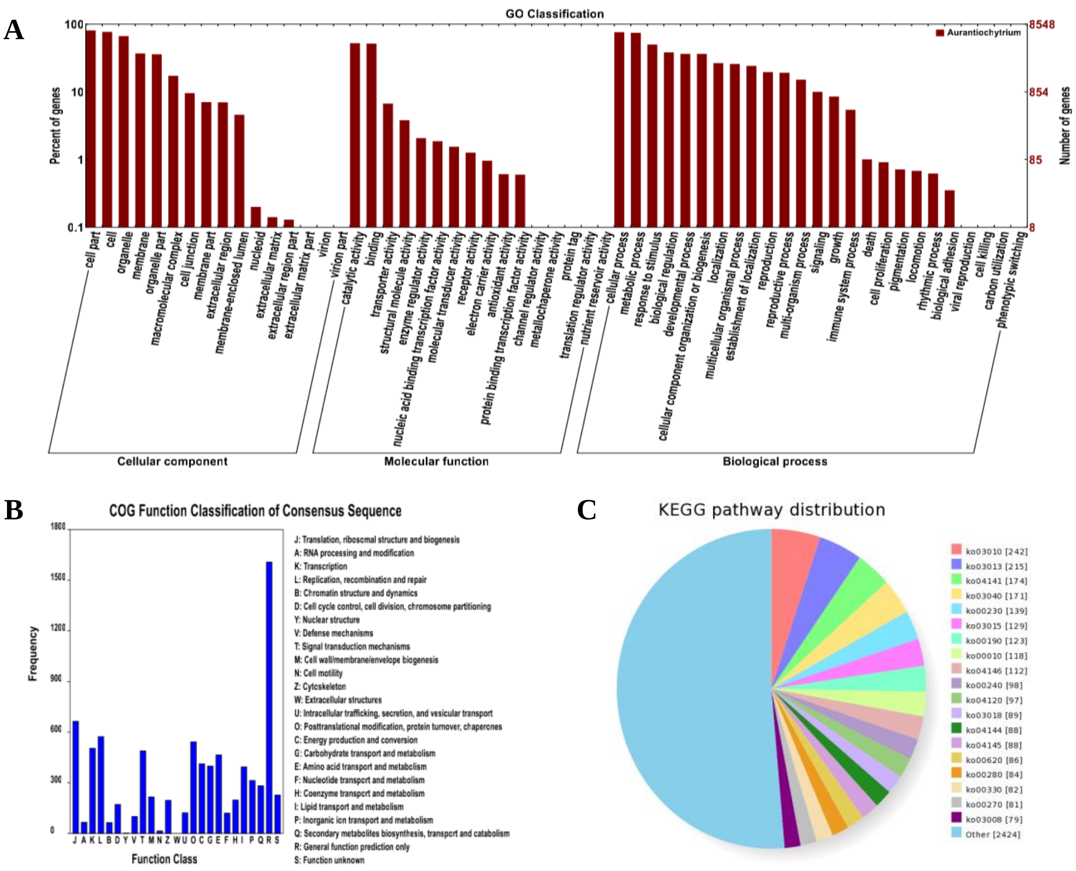

Figure S2. Functional annotation of assembled uni-genes in *Aurantiochytrium sp.* SK4. (A) GO classification, (B) COG classification, (C) KEGG pathway.

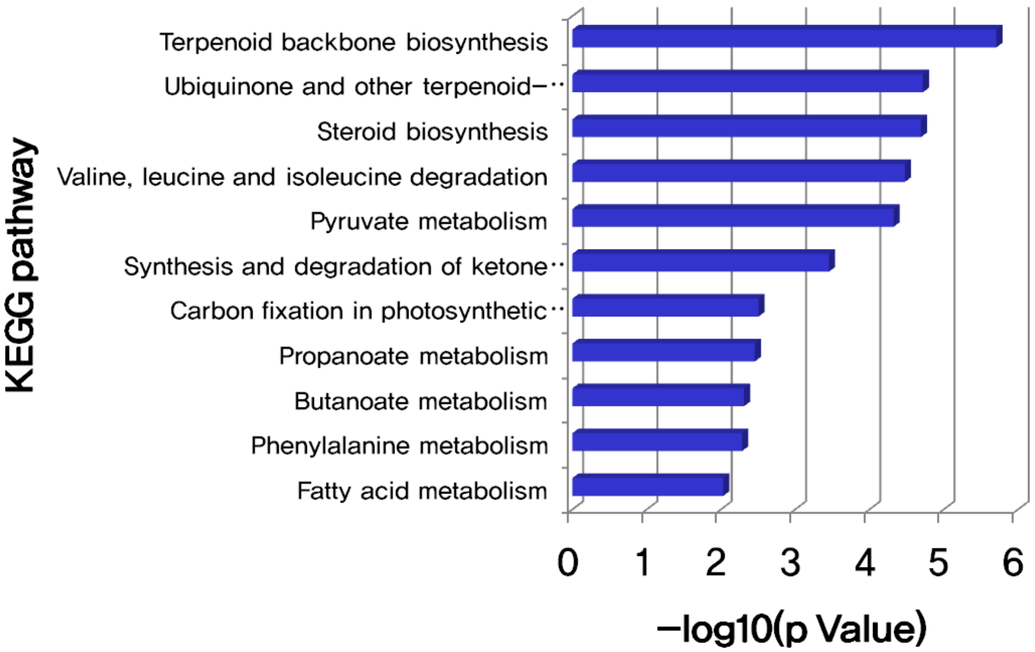

Figure S3. KEGG pathway enrichment analysis of differentially expressed genes (DEGs).

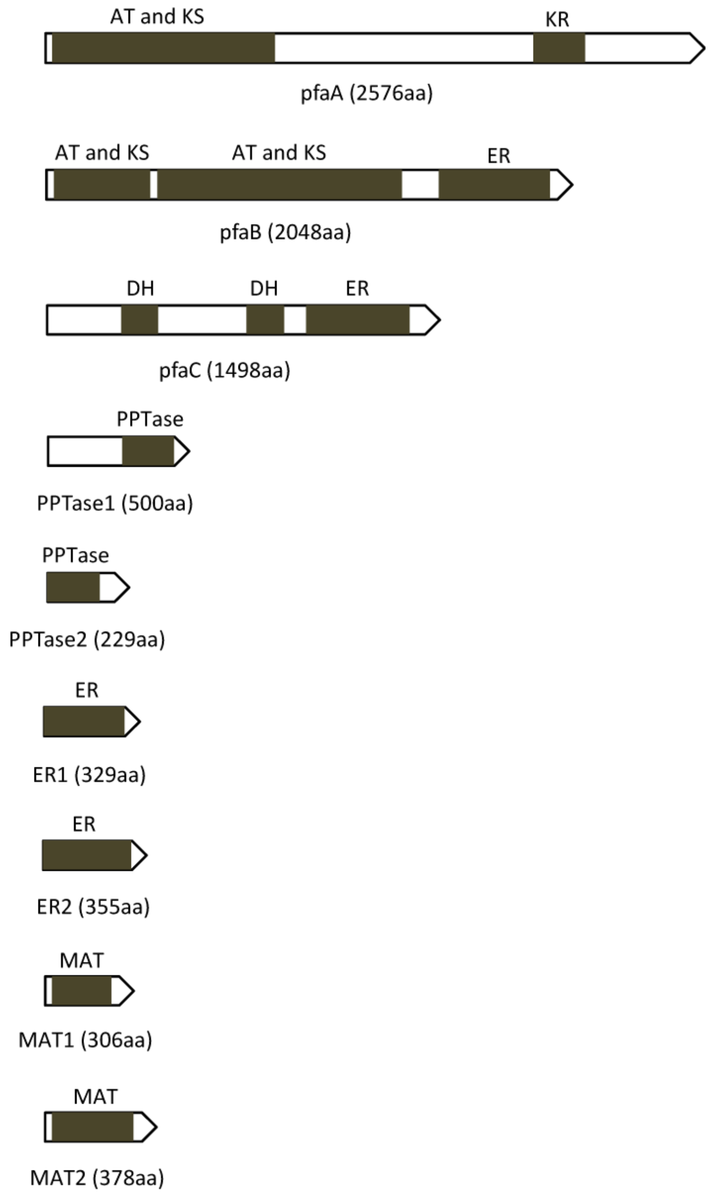

Figure S4. Genes encoding enzymes of the polyketide synthase (PKS pathway) in *Aurantiochytrium* sp.SK4. Dark gray areas indicate proposed enzymatic domains. KS, 3-ketoacyl synthase; KR, 3-ketoacyl-ACP reductase; ER, enoyl reducatase; DH, dehydrase/isomerase; PPTase, phosphopantetheine transferase; MAT, malonyl-CoA:ACP acyltransferase. aa, amino acid.

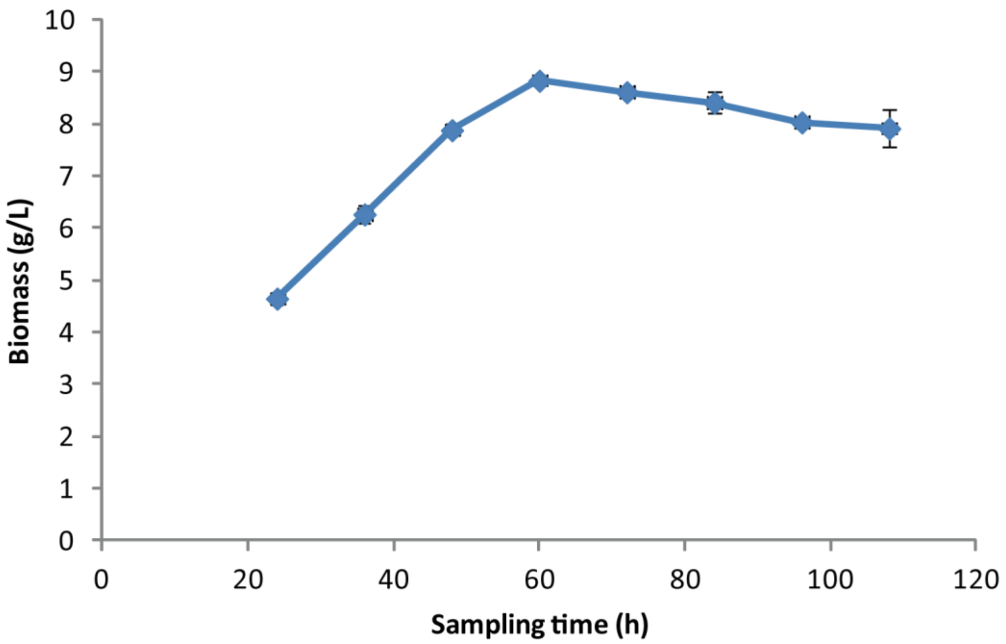

Figure S5. The growth curve of *Aurantiochytrium* sp.SK4 of Figure 1 and Figure 2.

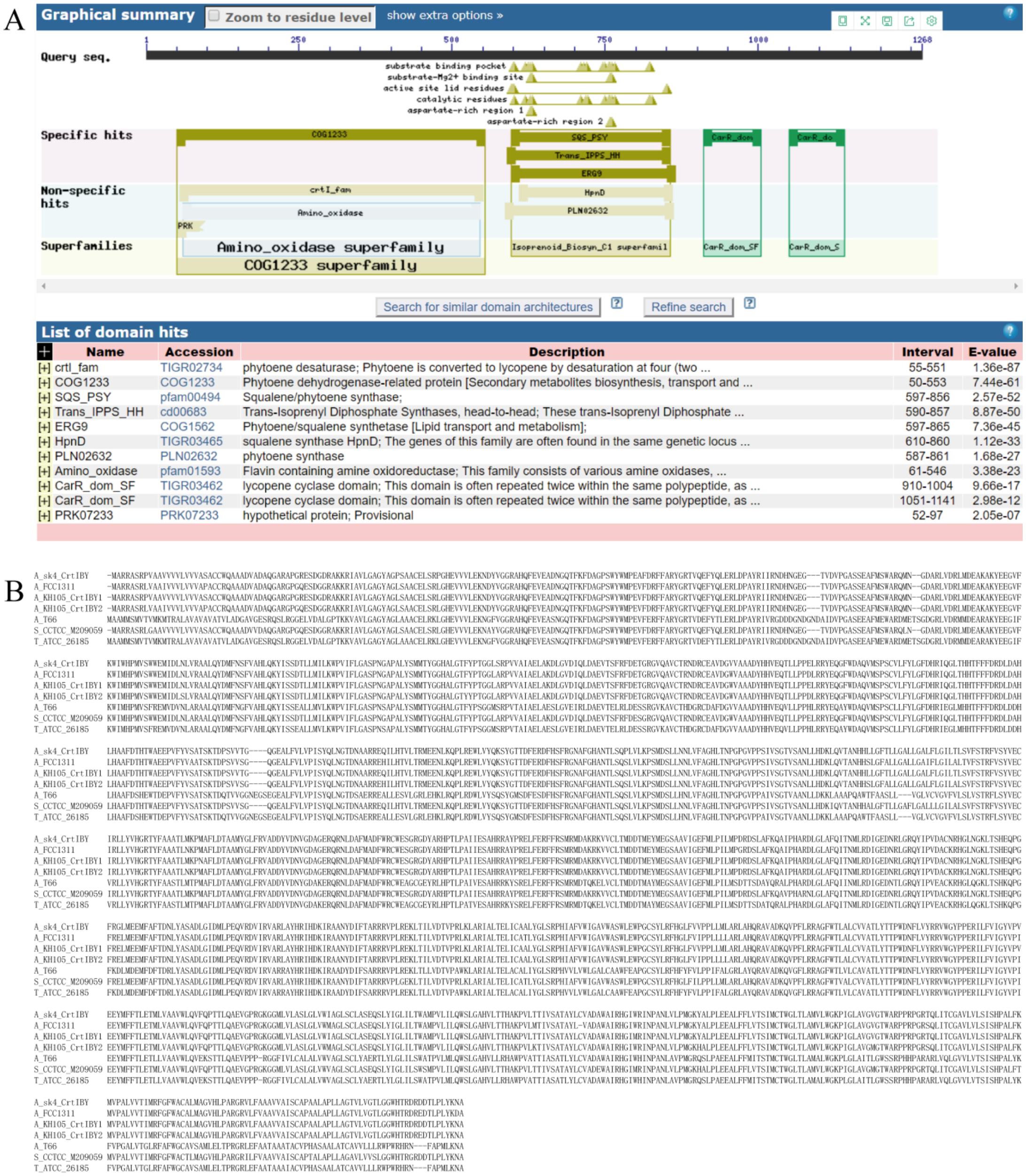

Figure S6. The conserved domains of CrtIBY and alignment of amino acid sequences of different CrtIBY. The conserved domains of CrtIBY from *Aurantiochytrium* sp.SK4 were analyzed by CDD/SPARCLE **(A)** and alignment of amino acid sequences of possible trifunctional β-carotene synthases, CrtIBY **(B)**. Sequences of CrtIBY of *Aurantiochytrium* sp. SK4 are compared with those of *Aurantiochytrium* sp. FCC1311, *Aurantiochytrium* sp. KH105, *Schizochytrium* sp. CCTCC M209059, *Aurantiochytrium* sp. T66, and *Thraustochytrium* sp. ATCC 26185.

Table S1

Comparison of *Aurantiochytrium* sp. SK4 genome statistics to other five algae and the *Arabidopsis thaliana* genome.

Organism	***Aurantiochytrium* sp. SK4**	***Arabidopsis thaliana***	***Chlamydomonas reinhardtii***	***Chlorella* sp.NC64A**	***Chromochloris zofingiensis***	***Coccomyxa subellipsoidea* C-169**	***Monoraphidium neglectum***
Sequenced genome size	49Mbp	119Mbp	107Mbp	42Mbp	58Mbp	49Mbp	67Mbp
Percent G+C in sequenced genome	56.7%	36%	64%	67%	51%	53%	65%
Coding sequence in sequenced genome	63.0%	28%	37%	32%	39%	25%	26%
Percent G+C in coding sequence	56.9%	44%	70%	69%	53%	61%	70%
Average number of exons	2.4	5.2	8.5	8.3	5.0	8.1	5.0
Average exon length	903nt	237nt	261nt	166nt	291nt	159nt	207nt
Percentage transcript with at least one intron	55.5%	76%	92%	98%	82%	94%	82%

Table S2

The expression of genes associated with carotenoids and fatty acid biosynthesis in transcriptome.

Pathway	Gene	RPKM	
		24h	96h
Astaxanthin pathway	*HMGS*	293.68	2.67
	HMGR	141.10	1.42
	MK	194.72	10.42
	PMK	21.56	12.61
	PPMD	60.02	1.11
	IDI	29.57	1.29
	CrtIBY	9.10	19.95
	CrtZ	3.87	12.66
	CrtO	14.94	28.95
FAS pathway	*Type 1 fatty acid synthase*	613.72	247.47
	Δ12 desaturase	377.4	2.0
	Δ5 desaturase	18.2	29.1
	ω-3 desaturase	0	0.6
	Δ4 desaturase	114.4	19.7
	Δ6 desaturase	0.7	0
	Δ9 desaturase	1.08	0
PKS pathway	*PKS pfaA*	754.56	1.95
	PKS pfaB	193.79	9.46
	PKS pfaC	381.54	4.38

Table S3

Contents of squalene in wild-type SK4 and the transformant AT26 at different stages.

	Sampling time	SK4	AT26
	48h	10.98 ± 0.13	0.34 ± 0.02
Squalene **content (mg × g^{-1} DW)**	72h	21.08 ± 0.06	0.41 ± 0.06
	96h	13.18 ± 0.05	0.64 ± 0.07

Data are shown as mean ± SD, n = 3.

Table S4

Primers Used for qRT-PCR and the detection of the p⁻–*VHb*–*ble*-2A-*IDI*-2A-*GPS* (VBIG).

Primer	Sequence
qRT-pcr-Actin-F	GAGGCCATGTTTCAGACCAT
qRT-pcr-Actin-R	ACGAGAGCCGTCATTTCTGT
qRT-pcr-HMGS-F	CGCCGGCGTCGACAGCAT
qRT-pcr-HMGS-R	GGGCACGGCGGGCAAGAC
qRT-pcr-HMGR-F	CCGGCGCAAAATGTCGAGTCT
qRT-pcr-HMGR-R	CCGCCGACAGTGCCAACCTC
qRT-pcr-MK-F	CCGCAACCACGAAATCCTCCAAAA
qRT-pcr-MK-R	CGAGAGCGCCGGCAGACTTG
qRT-pcr-PMK-F	GCCGTCTTTGCAGTTGTTGTTGATTC
qRT-pcr-PMK-R	GCCGCCGATCTTCACTCAGCAA
qRT-pcr-crtIBY-F	TGGTGACCTCGATCATGTGT
qRT-pcr-crtIBY-R	CGGCTCTACAGGTAATGAGT
qRT-pcr-FASⅠ-R	GAGAACGTCAGCACCTTTGC
qRT-pcr-FASⅠ-F	AGGCTCGAGAGAGCCTTGAC
qRT-pcr-pfaA-F	TGATCCCTTCGTGAATGACC
qRT-pcr-pfaA-R	GCTCGTTGTGGAACTGAAGG
qRT-pcr-pfaB-F	GTCATTCTGCCCCTCATCATCAACC
qRT-pcr-pfaB-R	GACTGCTTGGCGACCTGGTTCAC
qRT-pcr-pfaC-F	CCGCCCCATCCACGTCATCCTC
qRT-pcr-pfaC-R	CCGGACTGCTTGGCGACCTGGTT
VBIGF	GGCTTTGGCGATGACGGTATTG
VBIGR	CCCCTCCTCATCTCGTCCCTGT
